# Supplementary material for: Fabrication routes via projection stereolithography for 3D-printing of microfluidic geometries for nucleic acid amplification
Source: PLoS One. 2020 Oct 28;15(10):e0240237. doi: 10.1371/journal.pone.0240237 (PMC7592796; doi:10.1371/journal.pone.0240237)
Supplement: Attachment — Submitted filename: Response to Reviewers.docx [file pone.0240237.s002.docx]

**Rebuttal letter- Manuscript number PONE-D-20-09489**

Manuscript title: **“****Fabrication routes via projection stereolithography for 3D-printing of microfluidic geometries for nucleic acid amplification”**

Dear Reviewers/Editors,

we would like to thank you for your time to review the manuscript and the positive evaluation of our work. In general, we consider that the points made by the reviewers are correct and the revision work made to address them improved the manuscript. In the following paragraphs we quote each comment and explain how the revised version of the manuscript is modified around it. We further highlight the updates made in regards with data availability and according to PLOS requirements.

**Reviewer #1**
***1. “The miniaturized PCR device was claimed to be a microfluidic device. However, there was very little fluidic control demonstrated. The device looks more like a microwell to the reviewer. The authors shall specify more on the fluidic control, e.g. how was the PCR reagents delivered? Ideally, a better device design with fluidic control shall be demonstrated.”***

The reviewer makes a good point. We have changed the terminology to refer to a microfluidic chamber, and we discuss the potential for more complex microfluidic devices. In lines 146-149, it was further explained that the microchamber architecture was developed to implement stationary PCR thermocycling which is more reliable compared to the error prone flow-through method when employing resin 3D printed chips as shown in the work of Park and Park (*J. Park and H. Park, “Thermal cycling characteristics of a 3D-printed serpentine microchannel for DNA amplification by polymerase chain reaction,” Sensors and Actuators A: Physical, vol. 268, pp. 183-187, 2017*). In this way it was possible to characterise PCR inhibition, exclusively owed to material interactions rather than poor temperature control. In the case of flow-through PCR, poor fluidic control emerging from variable deviation levels of printed channel dimensions can result in poor temperature control that would lead to false interpretations of material PCR compatibility.

***2. “A related question is the level of complexity that DLP SLA printing would allow. The two-stage dip coating of the hydrophobic layer on chip may increase the production period and difficulty.”***

Complexity of channels achievable with the proposed methods is based on the melt viscosity of the wax, which determines the backpressure required for wax removal and feasibility depends on the bonding strength between the chip and the cap. In lines 354-356 it is mentioned that, the water-like melt viscosity of the low melting point paraffin wax, in combination with the unibody microchannel formed can deliver more complex 2.5D channels, such as heat exchanging architectures found in flow-through thermocycling setups. Although, such a geometry was not required in the present study, as it focuses specifically on the optimization of material properties to enable higher throughput platforms in the future. In regards with the second aspect of this comment, the coating processes for fluorination and glass coating lasted 2 hours and 1 hours respectively, as described in section 3.3 (line 368). These processes could be used for batches of chips, which was mentioned in the manuscript (line 375-376).

***3. “The authors claimed that “the system is qPCR ready by integration with a fluorescent microscope and qPCR DNA stains. However, this was not demonstrated experimentally in the manuscript.”***

The text has been amended. To highlight the potential of the proposed method of thermocycling for 3D printed chips, the following statement was added in the conclusions: “*From a system point-of-view, the proposed method of thermocycling shows promise for portability and modular integration of functionalities for diagnostic or research applications that utilize nucleic acid amplification technology.*”

**Reviewer #2:**
***1. “Defining the post-processing steps used in a 3-D printed device is useful but building standards/controls for those processing methods is really what is needed to extend this work beyond the custom chip and application described. Maybe add a comment or two about how this could be or was addressed so that it advances 3-D DLP-SLA printing for the field.”***

Within this paper we present well defined protocols for the processing methods, which can be adopted by researchers more broadly as the first stage towards standardised methods. Extending the work beyond prototyping volumes is largely a commercial decision, based on the price per chip. We have added to the discussion (lines 454-458-) and conclusions (lines 470-472) to consider this point. Further to this comment, and in regards with the creation of standards/controls for the post-processing methods presented, a new section has been added (2.2. Characterisation of printed resin interference with PCR). This section describes a preliminary experiment, that indirectly demonstrates that prolonged UV curing and hot water baths removes PCR inhibiting substances on the prints, facilitating the reaction in low SVR architectures (fluidics and milli fluidics). This method of post-processing was then adopted for the high SVR printed chip geometry, showing that although PCR inhibiting residuals are removed, inhibition due to adsorption becomes dominant in high SVR non-treated prints. This conclusion is drawn, since only hydrophobically modified chips produced amplicons consistent with the controls.

***2. “Consider adding a scale bar on Figure 4.”***

A scale bar was added, derived from the 3D CAD model assembly of the PCR thermocycler. Figure 4 was changed to Figure 5.

***3. “The three-chamber chip design reduced bubbles (downfall of any microfluidic device) but it was unclear if all three chambers hold 15 uL and are used in the PCR reaction or only the central chamber. Please clarify.”***

It was clarified that all three chambers hold 15 μL of PCR mixture, with each chamber accommodating approximately 5 μL (lines 163-165). Temperature sensing was performed only on the central chamber with the sensing element in contact with the fluid.

***4. “Line 284, I believe Figure 5, (d, f) is mislabeled and should be (a, d).”***

Figure 5 was changed to Figure 6. The figure was relabelled and the referencing in the text was updated.

***5. “Line 287, Figure 5 legend I believe the panels are mislabeled here as well. Shouldn’t it be (a,d) (b,e) and (c,f) with no i panel.”***

Figure 5 was changed to Figure 6. The figure was relabelled and the referencing in the text was updated.

***6. “Line 311 should reference Figure 7, currently there is an error message.”***

Cross-references were updated.

***7. “In Figure 7, the gel band alignments and the measurement of the 75 bp amplicon don’t always seem to line up well. I would suggest removing the mfg standard ladder image and label the bp bands on your agarose gel within the figure. Furthermore in panel c, it appears that the gel conditions changed (% agarose?, if so please also indicate in methods or legend) and the largest marker bands are no longer present within the gel but it actually the only one that does align with the marker image at left side of the panel.”***

Figure 7 was changed to Figure 8. The DNA ladder bands on the gel images were tagged individually. The text and the caption of the figure were further updated by clarifying that the gel representing the reactions performed on a non-treated chip (c panel) was 2% similar to a and b panels, but run for 20 minutes, instead of 45 for the modified printed chips that allowed for full ladder separation. This was for visualizing the faint amplicon generated on the non-treated chip before it was fully absorbed in the agarose gel at the end of the 45-minute run. Due to the early interruption of the agarose gel electrophoresis, the DNA ladder bands in Figure 8 (c), c are not fully separated but appear in groups for 766-100 and 75-25 base pair. This was confirmed by a few-minute additional rerun, which was added as an inset in Figure 8, (c) and is provided in the supporting information (S1_raw_images). However, the image comprehensively conveys the message of intense PCR inhibition owed to the reactivity of the non-treated printed polymer that creates suboptimal conditions, thereby disrupting the outcome of the reaction, providing a false negative.

**Data availability**

According to PLOS requirements for data availability, we have uploaded the raw data of the paper, including the blot/gel image data according to PLOS requirements, in the data repository of Newcastle university. Separate captions have also been added in the supporting information files. The supplementary information description and individual DOIs are mentioned below, as well as within the manuscript, under each figure and in the related section.

The supporting information files added are summarized below:

- **S1A_raw_images:** Raw agarose gel electrophoresis images for the mock PCR reactions presented in 2.2 and on-chip reactions presented in 3.5, Figure 5. (10.25405/data.ncl.12320501)
- **S1B_Fig_8_Amplicon_band­_intensity_plots:** Amplicon band intensity profiles of the on-chip reactions presented in 3.5, Figure 8. (10.25405/data.ncl.12320396)
- **S2A_Fig_6_SEM_contact_angle:** Raw SEM images and water contact angle visualizations of printed and functionalized disk specimens presented in 3.3, Figure 6. (10.25405/data.ncl.12320837)
- **S2B_contact_angle:** Contact angle values for all functionalized and non-functionalized printed disk specimens presented in 3.3, Figure 6. (10.25405/data.ncl.12320849)
- **S3_Fig_6_SEM_EDS:** Elemental analysis of the surface of printed and functionalized disk specimens presented in 3.3, Figure 6. (10.25405/data.ncl.12320861)
- **S4_ Fig_7_2_step_PCR-temperature_profile:** Temperature profile of the IR PCR system presented in 3.4, Figure 7 for a glass-coated 3D-printed chip ran a two-step PCR thermocycling protocol (10.25405/data.ncl.12320864).

We hope that we have successfully addressed the reviewers’ comments and clarified the presented data, giving a good insight into the versatile capabilities of high-resolution 3D-printing, specifically for microfluidics. We hope that the manuscript now meets the requirements for publication in PLOS one.

Yours sincerely,

Charalampos Tzivelekis
