# Supplementary material for: Fabrication routes via projection stereolithography for 3D-printing of microfluidic geometries for nucleic acid amplification
Source: PLoS One. 2020 Oct 28;15(10):e0240237. doi: 10.1371/journal.pone.0240237 (PMC7592796; doi:10.1371/journal.pone.0240237)
Supplement: Attachment — Submitted filename: Response to Reviewers.docx [file pone.0240237.s003.docx]

**Rebuttal letter- Manuscript number PONE-D-20-09489R1**

Manuscript title: **“****Fabrication routes *via* projection stereolithography for 3D-printing of microfluidic geometries for nucleic acid amplification”**

Dear Reviewers/Editors,

we would like to thank you for inviting us to submit a revised version of our manuscript and for the time and effort put into providing your feedback. We think that by clarifying the points raised during revision we further strengthened the manuscript. In the following paragraphs we quote the comments made by reviewer #3 and provide a point-by-point response, where we discuss the points and explain how these are addressed in the manuscript.

**Reviewer #3:**

*The original manuscript convincingly demonstrated the need for silanization of 3D printed PCR wells to obtain useful yields of PCR amplicons. The revised manuscript appropriately answered the reviewers’ questions. A few other minor questions could be answered by the authors in order to strengthen the revised manuscript further:*

**1. *One of the problems with SLA 3D printing is the volume shrinkage of the material used upon curing, which could induce high internal stress causing the deformation of material, and may eventually break the material. Did the authors observe or envision any such issues with the material they used? Did curing deformation affect the precision of the printed model?***

We have previously experienced deformations in microfluidic prints induced by over-curing with various resins. These usually result in flatness issues, especially for large print geometries. In this project, the utilized resin is commercially developed for high-definition prints of high thermal stability (e.g. moulds for injection moulding) and displays low deformation. By following the manufacturer’s instruction (*Formlabs, 2016*) for post-UV curing at approximately 80 mW/cm^2^*, we observed no distortion, at a level that would impair the device performance. The chips were designed to accommodate 15 μL fluid volumes, which were precisely loaded using a mechanical pipette. The reaction chamber withstood the pressure built during PCR thermocycling, verifying the good bonding quality, while the material remained rigid with no signs of deterioration. Flatness issues were not observed, potentially due to the small chip area and thickness which allowed a level of flexibility.

** chips were cured in a custom-built UV oven (355 nm, 0.003- 0.3 W/cm^2^, resolution 0.003 W/cm^2^)*

This point was addressed in the manuscript by adding the following statements:

**In 2.2, line 186-189:** “The utilized resin is commercially developed for high-definition prints of high thermal stability and minimum deformation and its main application is 3D printing of thermoforming tools. Layer exposure time was selected at 2 ± 0.1 seconds, so that optimal feature definition is achieved, as it was visually observed (Fig 1, 6)”**,**

**line 191-192:** “…according to the manufacturer instructions to achieve heat deflection temperature higher than 200°C”**,**

**in 3.2,** **line 382-386:** “By following the manufacturer instruction for post-UV curing with an 80 mW/cm^2^ light source, no distortion was observed at a level that would impair the performance of the printed device. The chips were designed to accommodate 15 μL fluid volumes, which were precisely loaded using a mechanical pipette. No flatness issues were observed, while the small chip area and thickness allowed for a level of flexibility.”,

**in 3.4, line 423-425:** “In terms of the material performance in thermocycling, the reaction chamber withstood the pressure built during PCR and the material remained rigid with no visual signs of deterioration.”

***2. Many resin materials with low viscosity have problems with biocompatibility, perhaps due to the residual photoinitiator or the degree of crosslinking of the material due to low viscosity. Can the authors comment on how the post-processing treatments and silane coupling agents affected biocompatibility?***

Biocompatibility in the wider sense was not part of this study. The focus was on developing a material suitable for nucleic acid amplification, in a sense that it does not interfere with the amplification process. In this context, the post processing treatments with ultraviolet (UV) curing and washing aim to reduce the amount of residuals (e.g. monomers, oligomers, photoinitator) which can be strong PCR inhibitors (*Inhibitory effect of common microfluidic materials on PCR outcome, 2012; Compatibility analysis of 3D printer resin for biological applications, 2016; Inhibition and Facilitation of Nucleic Acid Amplification, 1997*). UV curing achieves reduction of residuals through post-polymerization (i.e. consumption of the residuals in the polymer chain), while washing achieves reduction through removal with solvents of high monomer solubility (including water). Silane coupling agents created a hydrophobic passivated surface, reducing the potential for adsorption of components vital to the reaction, such as *Taq* polymerase (*Biochemical analysis and optimization of inhibition and adsorption phenomena in glass-silicon PCR-chips, 2003; Relief of Amplification Inhibition in PCR with Bovine Serum Albumin or T4 Gene 32 Protein, 1996*). Considering similar literature and our obtained results, the enzyme is the main adsorbed PCR component. The proposed hydrophobic treatments are appropriate for reducing biological adsorption in 3D printed microfluidics.

This comment was addressed in the manuscript by adding the following statements:

**In 3.1, line 358-361:** “UV curing reduced PCR inhibiting residuals by inducing their consumption into the polymerized material and increasing the degree of cross-linking. Water and isopropanol further dissolve print residuals to remove them from the printed surface.”,

**in 3.5, line 476-480:** “Silane treatments exploit the surface reactivity to create a hydrophobic passivated surface, which significantly limits the adsorption interface and level of reactivity. PCR components could not be adsorbed on the hydrophobic surface and the reaction was facilitated. Considering similar literature results, enzyme is the main adsorbed PCR component (*Biochemical analysis and optimization of inhibition and adsorption phenomena in glass-silicon PCR-chips, 2003; Relief of Amplification Inhibition in PCR with Bovine Serum Albumin or T4 Gene 32 Protein, 1996*). The proposed hydrophobic treatments are appropriate for reducing biological adsorption in 3D printed microfluidics.”

***3. Line 232, Figure 1 legend, change “positioning it” to “positioned”.***

***4. Line 315, Figure legend 4, add “for” to read it as “basis for further exploration”.***

***5. Line 423, Figure legend 7, change “denature” to “denaturation”.***

The figure legends were updated.

We think that the changes made reflect the reviewers’ comments and further clarify the presented data. We hope that the manuscript now meets the requirements for publication in PLOS one.

Yours sincerely,

Charalampos Tzivelekis
